# Lower Breast Cancer Risk among Women following the World Cancer Research Fund and American Institute for Cancer Research Lifestyle Recommendations: EpiGEICAM Case-Control Study

**DOI:** 10.1371/journal.pone.0126096

**Published:** 2015-05-15

**Authors:** Adela Castelló, Miguel Martín, Amparo Ruiz, Ana M. Casas, Jose M Baena-Cañada, Virginia Lope, Silvia Antolín, Pedro Sánchez, Manuel Ramos, Antonio Antón, Montserrat Muñoz, Begoña Bermejo, Ana De Juan-Ferré, Carlos Jara, José I Chacón, María A. Jimeno, Petra Rosado, Elena Díaz, Vicente Guillem, Ana Lluch, Eva Carrasco, Beatriz Pérez-Gómez, Jesús Vioque, Marina Pollán, EpiGEICAM researchers

**Affiliations:** 1 Cancer Epidemiology Unit, National Center for Epidemiology, Instituto de Salud Carlos III, Madrid, Spain; 2 Consortium for Biomedical Research in Epidemiology & Public Health (CIBERESP), Instituto de Salud Carlos III, Madrid, Spain; 3 Cancer Epidemiology Research Group, IIS Puerta de Hierro (IDIPHIM), Majadahonda, Madrid, Spain; 4 Medical Oncology Unit, Hospital Clínico Universitario San Carlos, Madrid, Spain; 5 Medical Oncology Unit, Instituto De Investigación Sanitaria Gregorio Marañón/ Universidad Complutense, Madrid, Spain; 6 Medical Oncology Unit, Instituto Valenciano de Oncología, Valencia, Spain; 7 Medical Oncology Unit, Hospital Virgen del Rocío, Sevilla, Spain; 8 Medical Oncology Unit, Hospital Puerta del Mar, Cádiz, Spain; 9 Medical Oncology Unit, Complejo Hospitalario Universitario A Coruña, A Coruña, Spain; 10 Medical Oncology Unit, Complejo Hospitalario de Jaén, Jaén, Spain; 11 Medical Oncology Unit, Centro Oncológico de Galicia, A Coruña, Spain; 12 Medical Oncology Unit, Hospital Universitario Miguel Servet, Zaragoza, Spain; 13 Medical Oncology Unit, Hospital Clinic I Provincial, Barcelona, Spain; 14 Medical Oncology Unit, Hospital Clínico, Valencia, Spain; 15 Medical Oncology Unit, Hospital Marqués de Valdecilla, Santander, Spain; 16 Medical Oncology Unit, Fundación Hospitalaria de Alcorcón, Alcorcón, Spain; 17 Medical Oncology Unit, Hospital Virgen de la Salud, Toledo, Spain; 18 Start-up Unit, Spanish Breast Cancer Research Group (GEICAM) Headquarters, San Sebastián De Los Reyes, Madrid, Spain; 19 Epidemiology Department, Universidad Miguel Hernandez, Sant Joan d'Alacant, Spain; University of Torino, ITALY

## Abstract

**Background:**

According to the “World Cancer Research Fund” and the “American Institute of Cancer Research” (WCRF/AICR) one in four cancer cases could be prevented through a healthy diet, weight control and physical activity.

**Objective:**

To explore the association between the WCRF/AICR recommendations and risk of breast cancer.

**Methods:**

During the period 2006 to 2011 we recruited 973 incident cases of breast cancer and *973* controls from *17* Spanish Regions. We constructed a score based on 9 of the WCRF/AICR recommendations for cancer prevention:: 1)Maintain adequate body weight; 2)Be physically active; 3)Limit the intake of high density foods; 4)Eat mostly plant foods; 5)Limit the intake of animal foods; 6)Limit alcohol intake; 7)Limit salt and salt preserved food intake; 8)Meet nutritional needs through diet; S1)Breastfeed infants exclusively up to 6 months. We explored its association with BC by menopausal status and by intrinsic tumor subtypes (ER+/PR+ & HER2-; HER2+; ER&PR-&HER2-) using conditional and multinomial logistic models respectively.

**Results:**

Our results point to a linear association between the degree of noncompliance and breast cancer risk. Taking women who met 6 or more recommendations as reference, those meeting less than 3 showed a three-fold excess risk (OR=2.98(CI95%:1.59-5.59)), especially for postmenopausal women (OR=3.60(CI95%:1.24;10.47)) and ER+/PR+&HER2- (OR=3.60(CI95%:1.84;7.05)) and HER2+ (OR=4.23(CI95%:1.66;10.78)) tumors. Noncompliance of recommendations regarding the consumption of foods and drinks that promote weight gain in premenopausal women (OR=2.24(CI95%:1.18;4.28); p for interaction=0.014) and triple negative tumors (OR=2.93(CI95%:1.12-7.63)); the intake of plant foods in postmenopausal women (OR=2.35(CI95%:1.24;4.44)) and triple negative tumors (OR=3.48(CI95%:1.46-8.31)); and the alcohol consumption in ER+/PR+&HER2- tumors (OR=1.52 (CI95%:1.06-2.19)) showed the strongest associations.

**Conclusion:**

Breast cancer prevention might be possible by following the “World Cancer Research Fund” and the “American Institute of Cancer Research” recommendations, even in settings like Spain, where a high percentage of women already comply with many of them.

## Introduction

Breast cancer (BC) is the most common cancer among women worldwide and, in spite of the continuous improvements in BC prognosis, this tumor constitutes the leading cause of cancer death among women in medium and high income countries [1–4]. Figures in Europe indicate that the absolute number of new diagnosis and deaths due to this disease continues to increase. Comparing the most recent estimates from 2008 and 2012, breast cancer incidence has risen from 421.000 [5] cases to 458.337 [6] in Europe with the subsequent personal and economic consequences. Recently published data reveals that, in 2009, the European health-care system expended €6.73billion in the diagnosis and treatment of BC, leading the ranking in terms of expenditure (13% of all cancer-related health-care costs) [7]. According to the scientific evidence, only a 5–10% of all cancer cases are due to genetic defects and the remaining 90–95% are attributable to environmental and lifestyle factors. Concretely, tobacco, diet, infection and obesity, contribute approximately 25–30%, 30–35%, 15–20% and 10–20% respectively, providing major opportunities for prevention [8]. A recent study about research gaps for BC prevention highlights, among the main critical needs, the implementation of sustainable changes in lifestyle based on diet, exercise and weight [9]. In this context, the “World Cancer Research Fund” (WCRF) and the “American Institute of Cancer Research” (AICR) issued in 2007, 8 general and 2 special recommendations on diet, physical activity and weight management for cancer prevention based on the available evidence[10,11]: 1)Maintain adequate body weight; 2)Be physically active; 3)Limit the intake of high density foods; 4)Eat mostly plant foods; 5)Limit the intake of animal foods; 6)Limit alcohol intake; 7)Limit salt and salt preserved food intake; 8)Meet nutritional needs through diet; S1)Breastfeed infants exclusively up to 6 months.

To our knowledge, only four studies have explored the specific association between these recommendations and BC risk [12–15] and none of them has classified the cases by tumor subtype considering hormonal receptors and the Human Epidermal Growth Factor Receptor 2 (HER2) status.

The objective of this study was to explore the association between WCRF/AICR recommendations and BC by menopausal status and pathological tumor subtype in Spain, a country traditionally characterized by healthy lifestyle habits.

## Methods

### EpiGEICAM case-control study

As we previously described [16], EpiGEICAM is a Spanish case-control study that recruited, between 2006 and 2011, *1017* incident cases of BC diagnosed in the Oncology departments of *23* hospitals members of the Spanish Breast Cancer Research Group (GEICAM: http://www.geicam.org) located in 9 of the *17* Spanish Regions. The participant oncologists invited the cases to participate in the moment of diagnosis. The inclusion criteria for cases were: age between 18 and 70 years old, agreement to participate and ability to understand and answer the questionnaire. Women previously diagnosed with breast cancer and women who were unable to answer the questionnaire due to health, language or educational issues were excluded. Each case was matched with a healthy control of similar age (± 5 years), selected from cases’ in-law relatives, friends, neighbors, or work colleagues residing in the same town.

Cases were sub classified by the following intrinsic subtypes based on local pathology reports: [17] 1) HER2- tumors (Estrogen Receptor (ER)+ or Progesterone Receptor (PR)+ with HER2-), 2) HER2+ tumors (HER2+ irrespective of ER or PR results); and 3) Triple negative tumors (ER-&PR-&HER2-). ER, PR and HER2 positivity were defined according to ASCO/CAP guidelines [18,19].

The EpiGEICAM study was approved by the Ethics Committees of all *23* participating hospitals (S4 Table). All participants signed an informed consent and patient information was anonymized and de-identified prior to analysis.

### Measurements

Cases and controls completed a structured and self-administered questionnaire collecting information on demographic and anthropometric characteristics, personal, family, obstetric and gynecologic history, physical activity and diet. Postmenopausal status was defined as absence of menstruation in the last 12 months. Dietary intake in the last five years was estimated using a *117*-item semi-quantitative food frequency questionnaire (FFQ) [20] adapted to and validated in different Spanish adult populations [21,22]. Upon agreement to participate, cases where invited to meet with the trained recruiters (nurses and other sanitary staff) that explained the study, proportionate the questionnaire and gave basic instructions to fill it in. In order to minimize the effect of recall bias, women were asked to respond within the following days and deliver the completed questionnaire in person within three months. They were also asked to bring their selected control in the next visit to follow the same process. The questionnaire was jointly reviewed by the participant and the interviewer in each center, who clarified those questions that the participant was not able to answer by herself.

The WCRF/AICR score was constructed following the 8 general and 2 special recommendations from WCRF/AICR report on food, nutrition and physical activity and the Continuous Update Project (CUP) for cancer prevention based on the available evidence [10,11]. Briefly, the score was based in 9 of the 10 recommendations: 1) Maintain adequate body weight; 2) Be physically active; 3) Limit the intake of high density foods; 4) Eat mostly plant foods; 5) Limit the intake of animal foods; 6) Limit alcohol intake; 7) Limit salt and salt preserved food intake; 8) Meet nutritional needs through diet; S1) Breastfeed infants exclusively up to 6 months. The special recommendation S2) for cancer survivors was not applicable to this population. A maximum score of 1 was assigned when the recommendation was fully met, an intermediate value of 0.5 when the recommendation was not far from being met and 0 points otherwise (Table 1). For the recommendations based in various subrecommendations, the final mark was calculated as the average of the subscores. The total mark was calculated as the sum of the scores in all 9 recommendations. Therefore, the WRCF/AICR score ranges from 0 to 9 and represents the minimum number of recommendations meet for each woman. The index was grouped in 5 categories [0–3[, [3–4[, [4–5[, [5,6 [and [6, 9]. The cut offs were defined as in Romaguera et al. [13] with the only exception of a wider last category. Categories “0–3” and “>3 to <4” were collapsed when the number of cases was smaller than 5.

**Table 1:** Operationalization of the WCRF/AICR in a score (0–9) using EpiGEICAM data.

WCRF/AICR recommendations	Personal recommendations	Operationalization^a^	Scoring
1) Maintain adequate body weight: Be as lean as possible without becoming underweight	**Self-reported BMI (in kg/m2) 18y**	18.5–24.9	1
	1a) Ensure that body weight through childhood and adolescence growth projects towards the lower end of normal BMI range at age 18y	25–29.9	0.5
		<18.5 or > = 30	0
	**BMI one year ago**	18.5–24.9	1
	1b) Maintain body weight within the normal range from age 18y	25–29.9	0.5
		<18.5 or > = 30	0
	**Weight gain per 10 years from 18yo**	≤2.5 Kg/10years	1
	1c) Avoid weight gain and increases in waist circumference throughout adulthood	2.5–5 Kg/10years	0.5
		>5Kg/10years	0
2) Be physically active as part of your everyday life	**Self-perception of physical activity during the last year**		
	2a) Be moderately physically active, equivalent to brisk walking, for > = 30min every day;	Vigorous	1
	2b) As fitness improves, aim for > = 60 min of moderate or for > = 30 min of vigorous physical activity every day;	Moderate	0.5
	2c) Limit sedentary habits such as watching television	Low	0
3) Limit consumption of energy-dense foods; avoid sugary drinks	**Energy-dense foods** ^**b**^	< = 125kcal/100 g	1
	3a) Consume energy-dense foods sparingly	125-175kcal/100 g	0.5
		>175lcal/100 g	0
	**Sugary drinks intake g** ^**c**^	0 g/d	1
	3b) Avoid sugary drinks	< = 250 g/d	0.5
		>250 g/d	0
	**Fast food intake g** ^**d**^	<18 g/d	1
	3c) Consume fast foods sparingly	18–42 g/d	0.5
		>42 g/d	0
4) Eat mostly foods of plant origin	**Fruits and vegetables** ^**e**^	> = 400 g	1
	4a) eat> = 5 portions/servings(> = 400 g) of a variety of non-starchy vegetables and fruit every day	200–400 g	0.5
		<200 g	0
	**Cereals, whole grain bread and legumes** ^**f**^	> = 64 g/d	1
	4b) Eat relatively unprocessed cereals (grains) and/or pulses(legumes) with every meal	24–64	0.5
		<24	0
	**White bread, pasta and rice** ^**g**^	<91 g/d	1
	4c) Limit refined starchy food	91–144 g/d	0.5
		> = 144	0
	4d) people who consume starchy roots or tubers as staples should also ensure sufficient intake of no starchy vegetables, fruit and pulses (legumes)	Not applicable to this population	
5) Limit intake of red meat and avoid processed meat	**Red (R) and processed (P) meat** ^**h**^	R+P<500g/wk and P<3g/d	1
	People who eat red meat should consume <500g/wk and very few, if any, processed meat	R+P<500g/wk and P 3-50g/d	0.5
		R+P> = 500g/wk or P> = 50 g/d	0
6) Limit alcoholic drinks	**Ethanol intake** ^**i**^	< = 10 g/d	1
	If alcoholic drinks are consumed, limit consumption to < = 1 drink/d	10–20 g/d	0.5
		> = 20 g/d	0
7) Limit consumption of salt and salt preserved food. Avoid moldy cereals (grains or pulses)	**Cold meat & salted/smoked fish** ^**j**^	< = 7 g/d	1
	7 a) Avoid slat-preserved, salted or salty foods. Preserve foods without using salt	7–22 g/d	0.5
		>22 g/d	0
	**Sodium**	<2.4	1
	7b) Limit consumption of processed foods with added salt to ensure an intake of sodium <2.4 g/d	2.4–3	0.5
		> = 3	0
	7c) Do not eat moldy cereals (grains) or pulses (legumes)	Insufficient data available	
8) Meet nutritional needs through diet alone	**Supplement use**	No	1
	8a) Dietary supplements are not recommended for cancer prevention	1 /d	0.5
		>1/d supplement	0
**WCRF/AICR especial recommendations**			
S1) Breastfeed infants exclusively up to 6 months	**Cumulative breastfeeding**	> = 6 mo	1
	Aim to breastfeed infants exclusively up to 6 months and continue with complementary feeding thereafter	>0 to <6 mo	0.5
		No breastfeeding	0
S2) Cancer survivors. Follow the recommendations for cancer prevention		Not applicable to this population	

^a^ Cutoffs provided in the “World Cancer Research Fund / American Institute for Cancer Research. Food, Nutrition, Physical Activity, and the Prevention of Cancer: a Global Perspective. Washington DC: AICR, 2007. In. 2007; 289–295.” or in the distribution of the data when the cut point was not specified (3c;4b;4c;7a;7b).

^b^ Energy intake for all foods considered.

^c^ Sugary drinks: juices and other sugared beverages.

^d^ Fast food: Fried potatoes, crisps, pizza, chicken and Serrano ham croquette, mayonnaise, tomato sauce, ketchup.

^e^ Fruits and vegetables: Orange, mandarin, banana, apple, pear, peach, nectarine, apricot, watermelon, melon, grapes, plums or prunes (dried or fresh), kiwi, spinach, chard, lettuce, endive, escarole, tomato, eggplant, zucchini, cucumber, pepper, artichoke, carrot, pumpkin, cooked cabbage, cauliflower or broccoli, onion, green beans, asparagus, corn and garlic.

^f^ Cereals, whole grain bread and legumes: Whole-grain bread and partial whole-grain bread, breakfast cereals and legumes.

^g^ Refined Grains: White-flour bread, rice, pasta.

^h^ Red and processed meat: Pork, beef, lamb, liver (beef, pork or chicken), entrails, hamburger, cold meat, sausages, bacon, pâte, foie-gras.

^i^ Alcohol: Measured as total ethanol intake coming from wine, beer and spirits.

^j^ Serrano ham and other cold meat and smoked and salt preserved fish.

### Statistical analysis

Smoking habit (<*1%*), age at first delivery (*5%*) and education (<*1%*) contained missing values. In order to obtain unbiased estimates of the effect of the recommendations using the information provided by all case-control pairs, missing values were imputed using multiple imputation with chained equations [23]. As explained in Royston et al [24] the chained equations method imputes missing values in different steps: Initially, all missing values are filled at random. The first variable with at least one missing value, smoking say, is then regressed on the other variables including those with missing values imputed at random in the initial step (BMI, physical activity, age at first delivery, education and age at menarche) and another set of potential explanatory variables that do not contain missing (menopausal status, age, number of children, hip and waist circumferences, bra size, calories, alcohol consumption and case/control status). The estimation is restricted to individuals with observed values for smoking and the missing values are replaced by simulated draws for the posterior predictive distribution of smoking. The next variable with missing values, say age at menarche, is regressed on all the other variables, included imputed values of smoking and restricting estimation to individuals with observed values for the variable to impute. Again, missing values for age at menarche are replaced by draws from the posterior predictive distribution. This process is repeated until a stable imputation is found for all values of all variables. Following this process we created five imputed data sets that were used for subsequent analyses. The final effect association is a weighted average of the effects found in these five datasets.

The association of the WCRF/AICR score with BC risk was evaluated using conditional logistic regression models with robust estimation of standard errors, both in categories and as a continuous term (considering the risk associated to one-unit decrease in the score). Same models where used to explore the association between the accomplishment of the individual recommendations and BC risk. All models included the following potential confounders: total calorie intake, smoking habit, age at first delivery, education, history of breast problems, family history of BC and menopausal status. Models for noncompliance of individual recommendations were also adjusted for the overall score obtained by adding up all the individual recommendations except the one under study. This approach was selected instead of adjusting for individual recommendations to avoid introducing collinearity in the models caused by the high dependence among them. Possible differences by menopausal status were assessed using interaction terms (1 df) between WRCF/AICR score/individual recommendations and menopausal status.

Multinomial logistic regression models were used to evaluate the association of the WCRF/AICR score/individual recommendations with each of the aforementioned intrinsic BC subtypes. These models were adjusted for age, hospital, and the same set of potential confounders described above.

The Wald test was used to compare the dose-response effect for each tumor subtype.

Assuming a causal relationship between the score WCRF/AICR and BC risk, the population attributable fraction (PAF%) was calculated using Levi's formula [25] to estimate the proportion of total cancer in this population that hypothetically would not have occurred if all participants were in the highest category of the score (6 or more recommendations met). Confidence intervals for PAF were computed using bootstrap with 1000 iterations.

Fractional polynomials were also used to explore the shape of the dose-response association between the score and BC risk [26].

Finally, a complete case analysis [23] was carried out for all models to check the validity of the imputation.

Analyses were performed in 2014 using STATA/MP 12.0 software.

### Results

After excluding *44* case-control pairs (*n = 88*) because of implausible reported energy intakes (<750 or >4500 kcal/day) [27] information in either the case or the control, final analyses were based on *973* cases-control pairs aged 22 to 71.

Compared to controls, BC cases seemed to accomplish less WCRF/AICR recommendations have higher age at first delivery, a lower education level, a larger proportion of breast problems and of family history of BC and a higher calorie intake (Table 2).

**Table 2:** Distribution WCRF/AICR score and individual recommendations and other baseline characteristics for cases and controls.

	Co/Ca	Pairs	Controls	Cases	P
**Score n(%)**	973/973	973			**<0.001**
6 to 9			265 (27%)	185 (19%)	
5 to <6			308 (32%)	291 (30%)	
4 to <5			266 (27%)	287 (29%)	
3 to <4			117 (12%)	172 (18%)	
0 to <3			17 (2%)	38 (4%)	
**Score mean(sd)**	973/973	973	5.21 (1.14)	4.93 (1.17)	**<0.004**
**Recommendations** Mean (sd)					
**1)** Maintain adequate body weight	912/902	852	0.63(0.32)	0.60(0.33)	**0.036**
**2)** Be physically active	907/893	834	0.45(0.39)	0.42(0.41)	0.085
**3)** Limit the intake of high density foods	973/973	973	0.64(0.23)	0.60(0.24)	**<0.001**
**4)** Eat mostly plant foods	973/973	973	0.61(0.23)	0.58(0.24)	**0.003**
**5)** Limit the intake of animal foods	973/973	973	0.32(0.29)	0.29(0.29)	**0.033**
**6)** Limit alcohol intake	973/973	973	0.88(0.28)	0.85(0.30)	**0.035**
**7)** Limit salt and salt preserved food intake	973/973	973	0.50(0.34)	0.45(0.34)	**0.001**
**8)** Meet nutritional needs through diet	973/973	973	0.69(0.38)	0.68(0.39)	0.376
**S1)** Breastfeed infants exclusively up to 6 months	816/800	681	0.50(0.44)	0.47(0.44)	0.246
**Smoking n(%)**	971/969	967			0.711
Never smoker			385 (40%)	395 (41%)	
Former smoker +6months			261 (27%)	267 (27%)	
Smoker or former smoker <6 months			325 (33%)	307 (32%)	
Unknown			2 (0%)	4 (0%)	
**Age at first delivery n(%)**	887/967	882			**<0.001**
<20			45 (5%)	49 (5%)	
20–24			208 (21%)	229 (24%)	
25–29			266 (27%)	258 (27%)	
>25			148 (15%)	216 (22%)	
Nulliparous			220 (23%)	215 (22%)	
Unknown			86 (9%)	6 (1%)	
**Education n(%)**	969/969	966			**0.003**
Primary school or less			159 (16%)	210 (22%)	
Secondary School			491 (50%)	503 (52%)	
University			319 (33%)	256 (26%)	
Unknown			4 (0%)	4 (0%)	
**History of breast problems n(%)**	973/973	973			**0.047**
No			796 (82%)	761 (78%)	
Yes			177 (18%)	212 (22%)	
**Family history of breast cancer n(%)**	973/973	973			**0.012**
None			782 (80%)	728 (75%)	
2nd degree			105 (11%)	129 (13%)	
1st degree			86 (9%)	116 (12%)	
**Menopausal Status n(%)**	973/973	973			0.084
Premenopausal			513 (53%)	551 (57%)	
Postmenopausal			460 (47%)	422 (43%)	
**Kcal intake mean(sd)**	973/973	973	1897 (628)	1990 (615)	**0.001**

Table 3 summarizes the results for the association between the WCRF/AICR scores and individual recommendations and BC risk by menopausal status. Despite the fact that the BC risk appeared to increase linearly with the decrease in the WCRF/AICR score, the categorical analyses showed the most significant risk for women with a score below 4. Women that accomplish only 3 recommendations showed a two-fold increased risk of BC than women in the upper category (OR = 2.09 (CI95%:1.46;2.99)) and women meeting less than 3 recommendations showed a three-fold increase in such a risk (OR_[0–3[vs[6–9]_ = 2.98 (CI95%:1.59–5.59)). The risk was higher in postmenopausal (OR_[0–3[vs[6–9]_ = 3.60 (CI95%:(1.24;10.47)) than in premenopausal women (OR_[0–3[vs[6–9]_ = 2.66 (CI95%:(1.23;5.76)), but confidence intervals overlap and the p-value for the interaction term in the continuous model was not statistically significant. The proportion of preventable cases of BC in this population by following 6 or more recommendations was estimated at around 30% for all women and also by menopausal status groups. Regarding specific items, diet related individual recommendations showed the strongest associations. In fact, noncompliance with recommendation 3 “Limit the intake of high density food” had an excess risk of 1.86 (CI95%:1.15;3.01), especially in premenopausal women (OR = 2.24 (CI95%: (1.18–4.28); p for interaction = 0.014), while a low intake of plant foods was also associated with BC (OR = 1.65 (CI95%:(1.08;2.57)), particularly among postmenopausal women (OR = 2.35 (CI95%:(1.24;4.44)), although the p-value for heterogeneity was not significant. The odds ratio of BC for women with alcohol consumption above the recommended was over 1.30 in all cases, however, none of these estimations showed statistical significance, probably given to the fact that most women (94% of controls and 95% of cases) meet totally or partially this specific recommendation.

**Table 3:** Association of WCRF/AICR score and individual recommendations with breast cancer risk by menopausal status.

	All women N = 1946		Premenopausal N = 1064		Postmenopausal N = 882		
WCRF/AICR score							
	CO/CA	OR^1^ (95%CI)	CO/CA	OR^1^ (95%CI)	CO/CA	OR^1^ (95%CI)	p-het
6 to 9	265/185	1	116/86	1	149/99	1	
5 to <6	308/291	1.35 (1.02;1.78)	154/168	1.51 (1.03;2.22)	154/123	1.20 (0.82;1.76)	
4 to <5	266/287	1.55 (1.17;2.06)	163/170	1.40 (0.96;2.05)	103/117	1.77 (1.18;2.65)	
3 to <4	117/172	2.09 (1.46;2.99)	69/106	2.13 (1.34;3.39)	48/66	2.04 (1.22;3.41)	
0 to <3	17/38	2.98 (1.59;5.59)	11/21	2.66 (1.23;5.76)	6/17	3.60 (1.24;10.47)	
**p-trend**		**<0.001**		**0.002**		**<0.001**	
**One unit decrease**		1.22 (1.11;1.34)		1.20 (1.06;1.36)		1.24 (1.10;1.41)	0.695
**Population Attributable Fraction (PAF%)** ^**2**^		32%(15%;50%)		33%(9%;56%)		34%(8%;59%)	
**Specific recommendations (Risk associated with the lack of compliance)**	**CO/CA** ^**3**^	**OR** ^**4**^ **(95%CI)**	**CO/CA**	**OR** ^**5**^ **(95%CI)**	**CO/CA**	**OR** ^**5**^ **(95%CI)**	**p-het**
**1)** Maintain adequate body weight	83/104	1.24 (0.91;1.70)	30/37	1.10 (0.72;1.68)	53/67	1.44 (0.90;2.30)	0.666
**2)** Be physically active	351/413	1.16 (0.91;1.48)	169/219	1.14 (0.82;1.58)	182/194	1.18 (0.83;1.66)	0.442
**3)** Limit the intake of high density foods	4/12	1.86 (1.15;3.01)	3/6	2.24 (1.18;4.28)	1/6	1.52 (0.80;2.89)	0.014
**4)** Eat mostly plant foods	13/16	1.65 (1.08;2.57)	10/10	1.22 (0.69;2.16)	3/6	2.35 (1.24;4.44)	0.489
**5)** Limit the intake of animal foods	412/458	1.04 (0.72;1.50)	239/279	1.21 (0.73;2.01)	173/179	0.91 (0.56;1.48)	0.456
**6)** Limit alcohol intake	61/79	1.35 (0.93;1.97)	28/38	1.39 (0.83;2.31)	33/41	1.32 (0. 780;2.22)	0.210
**7)** Limit salt and salt preserved food intake	189/220	1.22 (0.83;1.78)	104/125	1.18 (0.75;1.88)	85/95	1.26 (0.77;2.06)	0.474
**8)** Meet nutritional needs through diet	166/181	1.11 (0.86;1.44)	105/122	1.13 (0.80;1.59)	61/59	1.09 (0.73;1.62)	0.496
**S1)** Breastfeed infants exclusively up to 6 months	386/394	0.95 (0.70;1.27)	210/217	0.89 (0.61;1.30)	176/177	1.00 (0.69;1.45)	0.554

^1^ Adjusted for total calorie intake, smoking habit, age at first delivery, education, history of breast problems, family history of BC and menopausal status.

^2^ $\begin{array}{c} PAF=\frac{[PF_{\left[6-9\right]}\cdot \left(OR_{\left[6-9\right]}-1\right)]+[PF_{\left[5-6\right]}\cdot \left(OR_{\left[5-6\right]}-1\right)]+[PF_{\left[4-5\right]}\cdot \left(OR_{\left[4-5\right]}-1\right)]+[PF_{\left[3-4\right]}\cdot \left(OR_{\left[3-4\right]}-1\right)]+[PF_{\left[0-3\right]}\cdot \left(OR_{\left[0-3\right]}-1\right)}{1+[PF_{\left[6-9\right]}\cdot \left(OR_{\left[6-9\right]}-1\right)]+[PF_{\left[5-6\right]}\cdot \left(OR_{\left[5-6\right]}-1\right)]+[PF_{\left[4-5\right]}\cdot \left(OR_{\left[4-5\right]}-1\right)]+[PF_{\left[3-4\right]}\cdot \left(OR_{\left[3-4\right]}-1\right)]+[PF_{\left[0-3\right]}\cdot \left(OR_{\left[0-3\right]}-1\right)}•100\\ \begin{array}{c} \begin{array}{l} \text{PAF=}\;\text{Population}\;\text{Atributable}\;\text{Fraction}\;\\ \text{PF=Proportion}\;\text{of}\;\text{population}\;\text{in}\;\text{the}\;\text{specific}\;\text{exposure}\;\text{category}\\ \text{OR=}\;\text{Odds}\;\text{ratio}\;\text{for}\;\text{the}\;\text{especific}\;\text{exposure}\;\text{category} \end{array} \end{array} \end{array}$

^3^ Number of controls and cases that do not accomplish the specific recommendation.

^4^ OR per unit decrease (recommendation met vs not met). Adjusted for total calorie intake, smoking habit, age at first delivery, education, history of breast problems, family history of BC, menopausal status and score excluding the recommendation under study.

^5^ OR per unit decrease (recommendation met vs not met). Adjusted for total calorie intake, smoking habit, age at first delivery, education, history of breast problems, family history of BC and score excluding the recommendation under study.

Regarding the analyses by pathological subtype, even though no statistically significant differences were observed between subtypes, the increased risk for the lack of compliance with the WCRF/AICR recommendations was especially high for women with ER+/PR+&HER2- (OR_[0–3[vs[6–9]_ = 3.60 (CI95%:(1.84;7.05) and OR_[3–4[vs[6–9]_ = 2.18 (CI95%:(1.50;3.16)) and HER+ tumors (OR_[0–3[vs[6–9]_ = 4.23 (CI95%:(1.66;10.78)). Triple negative tumors were also associated with the WCRF/AICR score in the lower category (OR_[0–4[vs[6–9]_ = 2.32 (CI95%:(1.20;4.46)). The highest preventable effect of the WCRF/AICR guidelines was observed for ER+/PR+&HER2- (PAF95%CI: 35% (17%;53%)) and HER+ (PAF95%CI:34% (5%;62%)) tumors while such effect was not significant for the triple negative subtype. Again, for individual items, diet-related recommendations seemed to be the most important, particularly the consumption of foods and drinks that promote weight gain above the recommended which showed OR ranging from 1.68 for ER+/PR+/HER2- tumors to 2.93 for triple negative tumors, though the p-value for heterogeneity was not statistically significant. Low consumption of plant foods seemed to be specifically associated with triple negative tumors (OR = 3.48 (CI95%:(1.46–8.31)), with a p-value of heterogeneity of 0.148 while consumption of alcoholic drinks was only significantly associated with ER+/PR+&HER2- tumors (OR = 1.52 (CI95%:(1.06–2.19)) (Table 4).

**Table 4:** Association of WCRF/AICR score and individual recommendations with breast cancer risk by intrinsic tumor subtype.

		ER+/PR+&HER2- N = 653		HER+ N = 199		ER-&PR-&HER2- N = 119		
WCRF/AICR SCORE								
	CO	CA	OR^1^ (95%CI)	CA	OR^1^ (95%CI)	CA	OR^1^ (95%CI)	p-het
6 to 9	265	121	1	38	1	26	1	
5 to <6	308	193	1.35 (1.01;1.81)	59	1.30 (0.82;2.05)	39	1.22 (0.71;2.11)	
4 to <5	266	196	1.65 (1.21;2.24)	66	1.80 (1.13;2.88)	25	0.97 (0.52;1.80)	
3 to <4	117	118	2.18 (1.50;3.16)	28	1.64 (0.91;2.96)	26	2.32 (1.20;4.46)^2^	
0 to <3	17	26	3.60 (1.84;7.05)	9	4.23 (1.66;10.78)			
**p-trend**			**<0.001**		**0.004**		0.050	0.769
**One unit decrease**			1.26 (1.14;1.40)		1.20 (1.03;1.40)		1.20 (0.99;1.46)	0.701
**Population Attributable Fraction (PAF%)** ^**3**^			35%(17%;53%)		34%(5%;62%)		20%(-14%;55%)	
**SPECIFIC RECOMMENDATIONS(Risk associated with the lack of compliance)**	**CO** ^**4**^	**CA** ^**4**^	**OR** ^**5**^ **(95%CI)**	**CA** ^**4**^	**OR** ^**5**^ **(95%CI)**	**CA** ^**4**^	**OR** ^**5**^ **(95%CI)**	**p-het**
**1)** Maintain adequate body weight	83	64	1.13(0.81;1.57)	28	1.60 (0.97;2.63)	12	1.23 (0.65;2.30)	0.412
**2)** Be physically active	351	276	1.14 (0.88;1.48)	85	1.08 (0.73;1.61)	52	1.20 (0.74;1.97)	0.938
**3)** Limit the intake of high density foods	4	7	1.68 (1.01;2.80)	2	2.01 (0.93;4.35)	3	2.93 (1.12;7.63)	0.526
**4)** Eat mostly plant foods	13	13	1.47 (0.93;2.32)	2	1.40 (0.70;2.80)	1	3.48 (1.46;8.31)	0.148
**5)** Limit the intake of animal foods	412	319	1.11 (0.75;1.65)	91	0.96 (0.53;1.73)	48	0.76 (0.36;1.59)	0.590
**6)** Limit alcohol intake	61	58	1.52 (1.06;2.19)	14	1.27 (0.74;2.19)	7	0.93 (0.43;2.00)	0.414
**7)** Limit salt and salt preserved food intake	189	156	1.22 (0.82;1.82)	42	1.43 (0.78;2.64)	22	0.81 (0.38;1.72)	0.457
**8)** Meet nutritional needs through diet	166	136	1.29 (0.99;1.70)	32	1.02 (0.67;1.56)	13	0.88 (0.51;1.51)	0.272
**S1)** Breastfeed infants exclusively up to 6 months	386	266	1.02 (0.74;1.40)	79	0.85 (0.52;1.39)	49	1.23 (0.68;2.24)	0.591

^1^ Adjusted for total calorie intake, smoking habit, age at first delivery, education, history of breast problems, family history of BC, menopausal status, age and hospital.

^2^ OR and 95%CI for WRFC/AIRC score [0–4].

^3 $\begin{array}{c} PAF=\frac{[PF_{\left[6-9\right]}\cdot \left(OR_{\left[6-9\right]}-1\right)]+[PF_{\left[5-6\right]}\cdot \left(OR_{\left[5-6\right]}-1\right)]+[PF_{\left[4-5\right]}\cdot \left(OR_{\left[4-5\right]}-1\right)]+[PF_{\left[3-4\right]}\cdot \left(OR_{\left[3-4\right]}-1\right)]+[PF_{\left[0-3\right]}\cdot \left(OR_{\left[0-3\right]}-1\right)}{1+[PF_{\left[6-9\right]}\cdot \left(OR_{\left[6-9\right]}-1\right)]+[PF_{\left[5-6\right]}\cdot \left(OR_{\left[5-6\right]}-1\right)]+[PF_{\left[4-5\right]}\cdot \left(OR_{\left[4-5\right]}-1\right)]+[PF_{\left[3-4\right]}\cdot \left(OR_{\left[3-4\right]}-1\right)]+[PF_{\left[0-3\right]}\cdot \left(OR_{\left[0-3\right]}-1\right)}•100\\ \begin{array}{c} \begin{array}{l} \text{PAF=}\;\text{Population}\;\text{Atributable}\;\text{Fraction}\;\\ \text{PF=Proportion}\;\text{of}\;\text{population}\;\text{in}\;\text{the}\;\text{specific}\;\text{exposure}\;\text{category}\\ \text{OR=}\;\text{Odds}\;\text{ratio}\;\text{for}\;\text{the}\;\text{especific}\;\text{exposure}\;\text{category} \end{array} \end{array} \end{array}$^

^4^ Number of Controls (CO) and Cases (CA) that do not accomplish the specific recommendation.

^5^ OR per unit increase (recommendation met vs not met). Adjusted by total calorie intake, smoking habit, age at first delivery, education, history of breast problems, family history of BC, menopausal status, age, hospital and score excluding the recommendation under study.

The exploration of non-linear associations using fractional polynomials revealed that the linear model was the best fit when a continuous association was found (S1 Fig).

Sensitivity analyses gave similar results leading to the same conclusions (S1 and S2 Tables).

## Discussion

### Summary

Our results suggest that WCRF/AICR recommendations may help to prevent overall BC risk, especially among postmenopausal women and women with ER+/PR+&HER2- or HER+ tumor subtypes. Diet related individual recommendations seemed to be the factors more strongly associated with BC risk, especially a high consumption of high density foods or alcohol and the low intake of plant foods.

### Comparison with other studies

To our knowledge only four studies have explored the association between BC risk and the WCRF/AICR recommendations: two in the US, one in Canada and another using the European EPIC cohort, showing similar results to ours [12–15]. All of them report a significant linear negative trend for the association between the number of recommendations met and BC risk and two of them also identified women that meet 3 or less recommendations as the higher risk group [13,14]. Two of these studies also explored the specific relationship between individual recommendations and BC risk [12,15] and the authors found the strongest associations with the recommendations related to body fatness and food and alcohol intake that go in the same direction as ours.

Specific literature exploring the individual items that compose the WCRF/AICR score in relation with breast cancer, has pointed through a negative or non-significant effect of BMI or physical activity on the incidence of BC in premenopausal women and a positive association with postmenopausal breast cancer [10,11,28–30]. Our results, though not statistically significant, point in the same direction. Concerning diet and breast cancer, strong evidence is only available for the negative effect of alcohol consumption [10,11,31]. However some studies in low and medium income countries with greater dietetic variability suggested other interesting associations [31]. In this sense, various studies support our finding of a protective effect of plant foods intake against BC [32,33], particularly against RE-PR- tumors [34,35]. This is in agreement with the stronger effect we observed for triple negative tumors. Regarding the influence of foods and drinks that promote weight gain on BC development, to our knowledge, no specific studies have explored this association. The purpose of the third recommendation is to prevent cancer risk through a better control of body weight reducing the intake of energy-dense foods [10,11,36]. However, it is possible that the detrimental effect of this type of foods goes beyond the excess risk associated with an increase in body-weight, as our results suggest. Energy-dense foods not only include high-fat dietary products, but also highly sugared and processed foods that might have an effect on BC risk. The consumption of this type of food increases the risk especially in premenopausal women with higher adherence to a western-style diet [27].

The evidence of an association between consumption of red meat and processed food and BC is still weak [33,37,38], but it is in agreement with our results regarding recommendations 5 and 7. Despite the fact that alcohol is the only nutritional factor for which strong evidence of a positive association exists [10,11,31], we only identified a positive significant association with alcohol for women with ER+/PR+&HER2- tumors, even though results point through a positive association for BC in general. Our women did not report a high consumption of alcohol (only 79 cases and 61 controls reported an ethanol intake ≥20g/d) therefore differences between women in this case might be insufficient to obtain significant associations with the current sample size. Contrary to what is known for other tumors, vitamin supplementation has not been negatively associated with BC. In fact, some studies about supplementation with nutrients like vitamin C, D and E or calcium, to prevent BC have been published but the evidence is still insufficient to reach conclusions [39–42]. Finally, breast feeding appears to be a well-established protective factor for BC [10,11], but, we did not find a significant association in the analyses. In our sample only 28% of women did not breastfeed, being 97% of them nulliparous. These proportions might be too small to obtain significant results.

Regarding the potential preventability of the WCRF/AICR recommendations observed in our study, it is in concordance with the results published in the Policy and Action for Cancer Prevention Report [36] whose estimates for USA, UK, Brazil and China were 38%, 42%, 28% and 20% respectively.

### Limitations and Strengths

Recall bias is always a concern in case-control studies; however, the validity and reproducibility of FFQ was satisfactory [21,22] and the strength of the associations deemed it unlikely that our findings are a result of this bias. Secondly, statistical power was limited in the subgroup analyses by intrinsic tumor subtype and therefore the results should be interpreted with caution. On the other hand, the matching design resulted in closely related cases and controls which would bias the OR towards the null effect. In spite of these limitations, we were able to detect a consistent dose-response gradient for the association between BC and WCRF/AICR score, even in the stratified and subgroup analyses.

Except for the cases of the specific subrecommendations related to moldy cereals or pulses, we were able to operationalize all general and specific WCRF/AICR recommendations applicable to this population. No previous studies have been able to operationalize all the recommendations with their data and only one was able to explore the individual association between BC risk and 6 out of the 9 recommendations. On the other hand, this is the first study that explores such associations by menopausal status and BC pathological subtype including ER, PR and HER2 status.

Finally, Spain is a country that has traditionally maintained healthy dietary habits. In fact, almost 60% of the control population met 5 or more recommendations and 90% of our women accomplish somehow the most important recommendations on food (R1 and R4) and alcohol consumption (R6) (S4 Table). However, our results suggest that our women can still benefit from a greater adherence to the WCRF/AICR recommendations.

## Conclusions

BC prevention might be possible by following the WRCF/AICR recommendations, even in settings like Spain, where a high percentage of women already comply with many of them. Despite the fact that especial benefit can be obtained by avoiding the consumption of foods and drinks that promote weight gain, limiting alcohol intake and increasing the consumption of plant foods, our results indicate that a good level of satisfaction with most of the recommendations is more important than any single recommendation.

## Supporting Information

S1 Fig Graphical representation of best polynomial fit for all women and stratifying by menopausal status and type of tumor including p-value for departure from linearity. (PDF)

S1 Table Association of WCRF/AICR score with breast cancer risk by menopausal status. Complete case analysis.
(DOCX)

S2 Table Association of WCRF/AICR score with breast cancer risk by intrinsic tumor subtype. Complete case analysis.
(DOCX)

S3 Table Number and percentage of recommendations accomplished by cases and controls. (DOCX)

S4 Table Names of all approving ethics committees. (DOCX)
