# Supplementary material for: Lower Breast Cancer Risk among Women following the World Cancer Research Fund and American Institute for Cancer Research Lifestyle Recommendations: EpiGEICAM Case-Control Study
Source: PLoS One. 2015 May 15;10(5):e0126096. doi: 10.1371/journal.pone.0126096 (PMC4433351; doi:10.1371/journal.pone.0126096)

Lower breast cancer risk among women following WCRF/AICR lifestyle recommendations: EpiGEICAM case-control study. Breast Cancer Research and Treatment. Adela Castelló, Miguel Martín, Amparo Ruiz, Ana M. Casas, Jose M Baena-Cañada, Virginia Lope, Silvia Antolín, Pedro Sánchez, Manuel Ramos, Antonio Antón, Montserrat Muñoz, Begoña Bermejo, Ana de Juan-Ferré, Carlos Jara, José I Chacón, María A. Jimeno, Petra Rosado, Elena Díaz, Vicente Guillem, Ana Lluch, Eva Carrasco, Beatriz Pérez-Gómez, Jesús Vioque, Marina Pollán on behalf of GEICAM researchers.

CORRESPONDING AUTHOR: Dr. Marina Pollán.Cancer Epidemiology Unit, National Center for Epidemiology. Instituto de Salud Carlos III.e-mail: mpollan@isciii.es

S1 Fig: Graphical representation of best polynomial fit for all women and stratifying by menopausal status and type of tumor including p-value for departure from linearity.

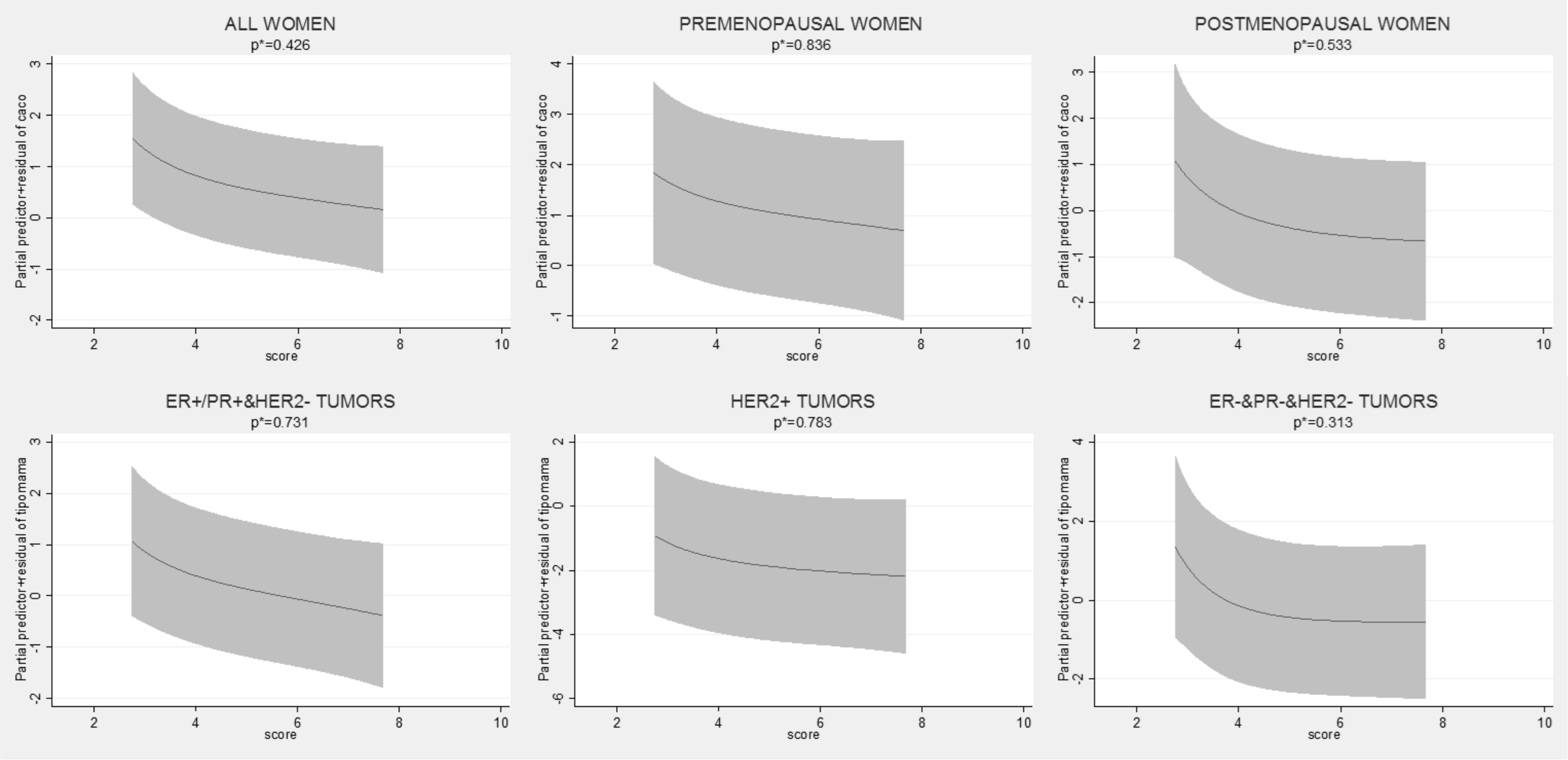

Supplement: S1 Fig — (PDF) [file pone.0126096.s001.pdf]
