# Supplementary material for: Lower Breast Cancer Risk among Women following the World Cancer Research Fund and American Institute for Cancer Research Lifestyle Recommendations: EpiGEICAM Case-Control Study
Source: PLoS One. 2015 May 15;10(5):e0126096. doi: 10.1371/journal.pone.0126096 (PMC4433351; doi:10.1371/journal.pone.0126096)
Supplement: S2 Table — Complete case analysis. (DOCX) [file pone.0126096.s003.docx]

**S2 Table:** Association of WCRF/AICR score with breast cancer risk by intrinsic tumor subtype. Complete case analysis.

|  |  | **ER+/PR+&HER2-**  **N=653** | | **HER+**  **N=199** | | **ER-&PR-&HER2-**  **N=119** | |  |
| --- | --- | --- | --- | --- | --- | --- | --- | --- |
| **WCRF/AICR score** |  |  |  |  |  |  |  |  |
|  | **CO** | **CA** | **OR^1^ (95%CI)** | **CA** | **OR^1^ (95%CI)** | **CA** | **OR^1^ (95%CI)** | **p-het** |
| **[6-9**] | 234 | 118 | 1 | 38 | 1 | 26 | 1 |  |
| **[5-6[** | 285 | 189 | 1.33 (0.98;1.79) | 58 | 1.25 (0.79;1.98) | 38 | 1.17 (0.67;2.03) |  |
| **[4-5[** | 237 | 193 | 1.69 (1.23;2.32) | 66 | 1.83 (1.14;2.93) | 25 | 1.02 (0.55;1.90) |  |
| **[3-4[** | 109 | 118 | 2.19 (1.49;3.20) | 27 | 1.57 (0.86;2.85) | 26 | 2.35 (1.21;4.56)^2^ |  |
| **[0-3[** | 17 | 25 | 3.37 (1.71;6.65) | 9 | 4.05 (1.59;10.34) |  | -- |  |
| **p-trend** |  |  | 0.000 |  | 0.004 |  | 0.037 |  |
| **One unit decrease** |  |  | 1.27 (1.14;1.40) |  | 1.20 (1.03;1.40) |  | 1.22 (1.00;1.48) | 0.781 |
| **Population Attributable Fraction (PAF%)^3^** |  |  | 35%(19%,51%) |  | 33%(6%;59%) |  | 20%(-15%;56%) |  |
| **Specific recommendations**  **(Risk associated with the lack of compliance)** | **CO^4^** | **CA^4^** | **OR^5^(95%CI)** | **CA^4^** | **OR^5^(95%CI)** | **CA^4^** | **OR^5^(95%CI)** | **p-het** |
| **1) Body fatness** | 71 | 60 | 1.15 (0.82;1.59) | 28 | 1.62 (0.99;2.67) | 12 | 1.27 (0.68;2.37) | 0.418 |
| **2) Physical activity** | 323 | 267 | 1.19 (0.92;1.54) | 84 | 1.12 (0.76;1.67) | 51 | 1.28 (0.78;2.09) | 0.914 |
| **3) Foods and drinks that promote weight gain** | 3 | 7 | 1.58 (0.95;2.62) | 2 | 1.88 (0.87;4.06) | 3 | 2.69 (1.03;7.00) | 0.555 |
| **4) Plant foods** | 11 | 13 | 1.46 (0.93;2.30) | 2 | 1.40 (0.70;2.80) | 1 | 3.38 (1.42;8.06) | 0.165 |
| **5) Animal foods** | 369 | 316 | 1.11 (0.75;1.64) | 89 | 0.96 (0.54;1.73) | 48 | 0.77 (0.37;1.60) | 0.609 |
| **6) Alcoholic drinks** | 56 | 57 | 1.52 (1.06;2.17) | 13 | 1.26 (0.73;2.16) | 7 | 0.92 (0.43;1.98) | 0.407 |
| **7) Preservation, processing and preparation** | 170 | 155 | 1.23 (0.83;1.84) | 42 | 1.45 (0.79;2.68) | 22 | 0.83 (0.39;1.76) | 0.465 |
| **8) Dietary supplements** | 166 | 136 | 1.34 (1.02;1.76) | 32 | 1.05 (0.69;1.61) | 13 | 0.93 (0.54;1.59) | 0.292 |
| **S1) Breastfeeding** | 359 | 262 | 1.01 (0.73;1.39) | 77 | 0.85 (0.52;1.39) | 48 | 1.24 (0.68;2.26) | 0.577 |

^1^ Adjusted by total calorie intake, smoking habit, age at first delivery, education, history of breast problems, family history of BC, menopausal status, age and hospital.

^2^ OR and 95%CI for WRFC/AIRC score [0-4[.

^3^ $\begin{matrix} PAF=\frac{[PF_{\left[6-9\right]}\cdot\left(OR_{\left[6-9\right]}-1\right)\left]+\right[PF_{\left[5-6\right]}\cdot\left(OR_{\left[5-6\right]}-1\right)]+[PF_{\left[4-5\right]}\cdot\left(OR_{\left[4-5\right]}-1\right)\left]+\right[PF_{\left[3-4\right]}\cdot\left(OR_{\left[3-4\right]}-1\right)]+[PF_{\left[0-3\right]}\cdot\left(OR_{\left[0-3\right]}-1\right)}{1+[PF_{\left[6-9\right]}\cdot\left(OR_{\left[6-9\right]}-1\right)\left]+\right[PF_{\left[5-6\right]}\cdot\left(OR_{\left[5-6\right]}-1\right)]+[PF_{\left[4-5\right]}\cdot\left(OR_{\left[4-5\right]}-1\right)\left]+\right[PF_{\left[3-4\right]}\cdot\left(OR_{\left[3-4\right]}-1\right)]+[PF_{\left[0-3\right]}\cdot\left(OR_{\left[0-3\right]}-1\right)}\bullet100 \\ \begin{matrix} \begin{matrix} \text{PAF= Population Atributable Fraction } \\ \text{PF=Proportion of population in the specific exposure category} \\ \text{OR= Odds ratio for the especific exposure category} \end{matrix} \\ \end{matrix} \end{matrix}$

^4^ Number of Controls (CO) and Cases (CA) that do not accomplish the specific recommendation.

^5^ OR per unit increase (recommendation met vs not met). Adjusted by total calorie intake, smoking habit, age at first delivery, education, history of breast problems, family history of BC, menopausal status, age, hospital and score excluding the recommendation under study.
