# Supplementary material for: Lower Breast Cancer Risk among Women following the World Cancer Research Fund and American Institute for Cancer Research Lifestyle Recommendations: EpiGEICAM Case-Control Study
Source: PLoS One. 2015 May 15;10(5):e0126096. doi: 10.1371/journal.pone.0126096 (PMC4433351; doi:10.1371/journal.pone.0126096)
Supplement: S3 Table — (DOCX) [file pone.0126096.s004.docx]

**S3 Table:** Number and percentage of recommendations accomplished by cases and controls.

|  | Not met^*^ | Not far from met^*^ | Met^*^ | Missing |
| --- | --- | --- | --- | --- |
|  | Controls/Cases  n(%) | Controls/Cases  n(%) | Controls/Cases  n(%) | Controls/Cases  n(%) |
| **1) Body fatness** | 67(7%)/ 80(8%) | 461(47%)/459(47%) | 384(39%)/363(37%) | 61(6%)/ 71(7%) |
| **2) Physical activity** | 285(29%)/333(34%) | 362(37%)/299(31%) | 260(27%)/261(27%) | 66(7%)/ 80(8%) |
| **3) Foods and drinks that promote weight gain** | 44(5%)/ 70(7%) | 605(62%)/613(63%) | 324(33%)/290(30%) | 0(0%)/0(0%) |
| **4) Plant foods** | 55(6%)/ 78(8%) | 643(66%)/665(68%) | 275(28%)/230(24%) | 0(0%)/0(0%) |
| **5) Animal foods** | 412(42%)/458(47%) | 506(52%)/469(48%) | 55(6%)/ 46(5%) | 0(0%)/0(0%) |
| **6) Alcoholic drinks** | 61(6%)/ 79(8%) | 107(11%)/125(13%) | 805(83%)/769(79%) | 0(0%)/0(0%) |
| **7) Preservation, processing and preparation** | 189(19%)/220(23%) | 401(41%)/423(43%) | 383(39%)/330(34%) | 0(0%)/0(0%) |
| **8) Dietary supplements** | 166(17%)/181(19%) | 265(27%)/265(27%) | 542(56%)/527(54%) | 0(0%)/0(0%) |
| **S1) Breastfeeding** | 229(24%)/221(23%) | 205(21%)/234(24%) | 382(39%)/345(35%) | 157(16%)/173(18%) |

^*^Defined as: Not met= score in recommendation <0.25; Not far from met = score in recommendation 0.25-075; Met= score in recommendation ≥0.75
