# Supplementary material for: Lower Breast Cancer Risk among Women following the World Cancer Research Fund and American Institute for Cancer Research Lifestyle Recommendations: EpiGEICAM Case-Control Study
Source: PLoS One. 2015 May 15;10(5):e0126096. doi: 10.1371/journal.pone.0126096 (PMC4433351; doi:10.1371/journal.pone.0126096)
Supplement: S4 Table — (DOCX) [file pone.0126096.s005.docx]

**S4 Table: Names of all approving ethics committees.**

| **approving ethics committees** |
| --- |
| CEIC Fundación Instituto Valenciano de Oncología |
| CEIC del Hospital Universitario Virgen del Rocío de Sevilla |
| CEIC del Hospital Universitario Puerta del Mar |
| CEIC de Galicia |
| CEIC Área 7 - Hospital Clínico San Carlos de Madrid |
| CEIC Hospital Clinic de Barcelona |
| CEIC Hospital Clínico Universitario de Valencia |
| CEIC de Cantabria |
| CEIC Área 8 - Fundación Hospital Alcorcón |
| CEIC de Jaén |
| CEIC de Aragon - CEICA |
| CEIC Complejo Hospitalario de Toledo |
| CEIC Hospital Mutua de Terrassa |
| CEIC Hospital Universitari de Bellvitge |
| CEIC Hospital General Universitario de Alicante |
| CEIC Hospital Virgen de los Lirios de Alcoy |
| CEIC Hospital Universitari de Girona Dr. Josep Trueta |
| CEIC Hospital Mutua de Terrassa |
| CEIC Hospital Universitari Arnau de Vilanova de Lleida |
| CEIC Área de Salud de Burgos y Soria |
| CEIC Fundacio d.Osona per a la Recerca i Educacio Sanitaries - FORES |
